# Community-Level Health Interventions are Crucial in the Post-COVID-19 Era: Lessons from Africa’s Proactive Public Health Policy Interventions

**DOI:** 10.1007/s41463-022-00127-3

**Published:** 2022-06-28

**Authors:** Frederick Ahen

**Affiliations:** Faculty of Social Sciences and Business Studies, Business School, University of Eastern Finland, Yliopistonranta 1, 70210 Kuopio, Finland

**Keywords:** Africa, COVID-19, Political will, Pre-emptive interventions, Public health inequality, Vaccine nationalism

## Abstract

Measured against the gloomy pre-COVID-19 predictions, Africa has fared far better than most regions in managing the pandemic. This much, however, has received less attention. This paper answers the question: how have the new rituals of self determination in public health affected the successful management of COVID-19 in Africa, and how can the continent and the rest of the world build on such models/lessons in the post-pandemic era? I employ emancipatory theorising in reviewing literature on approaches to governance of COVID-19. The rationale is to empower the grassroots and to accentuate the urgency for a decolonized local ownership of the governance of all public health crises. I argue that while traditional international cooperation is necessary for additional resource and expertise from the global North for sustainable health, the political will of Southern governments remains fundamental for any extraordinary success due to its grassroots/community orientation towards non-pharmaceutical interventions and initial pre-emptive rituals. The novelty in this paper is that it lays bare the ignored African responses and lessons and reveals how to harness protective communitarian ethos in solving future crises. The paper further provides population health as an ‘immune system’ policy framework for explaining and predicting how a scientific and human-centrered grassroots leadership can yield optimal outcomes in any future crisis.

## Background and Arguments

The world is still not out of the woods. Countless uncertainties remain with the governance of the COVID-19 pandemic (Ahen 2021; Wamai et al. 2021; Beaton et al. 2021). The challenge of susbtantive health inequalities keeps increasing in complexity (Mehtar et al. 2020; Ottersen et al. 2014). This humanistic policy-oriented paper punctually arrives during an unprecedented global epidemiological crisis with a two-fold purpose:

(i) To articulate the antecedents of grassroots rituals of self-determination in public health governance, the key success factors in the management of COVID-19 in Africa and how such blueprint can be built upon to further the progress made thus far.
(ii) To supply a conceptual framework for a scenario-based analysis and decision making about complex sustainable health problems, considering the relevant structural and social determinants of health, and how the different operational factors produce specific outcomes based on the nature of scientific, political and resource inputs.

The paper answers the following questions: how have the new rituals of public health self-determination affected the successful management of COVID-19 in Africa and how can the continent and the rest of the world build on such models/lessons in the post-pandemic era? The objective here is to initiate a paradigm shift in the framing and communication of Africa’s public health policy discourses. This stems from the observation that if African governments are consistently discredited and not recognised for any improved health outcomes, it reads a negative signal into subsequent actions that says: “No matter what we do, we will not be acknowledged but outsiders such as international non-governmental organizations (INGOs), the World Health Organization (WHO) and philanthropists will be praised instead. We will be seen as eternally inept. Therefore, we will be complacent with mediocre performance.” By contrast, if they are credited for their efforts, it will serve as further impetus for doing more even with less resources as in this case of COVID-19 outbreak. Despite surging infections around the world, Africa is still the continent least affected by the Severe Acute Respiratory Syndrome Coronavirus 2 (SARS-CoV-2) causing the disease COVID-19, albeit disputed in some quarters (Wamai et al. 2021; WHO 2021; El-Sadr and Justman 2020). As at 12^th^ of April 2022, the total global cumulative cases stood at 497,960,492 (Africa 8,614,444) and total cumulative deaths at 6,181,850 (Africa 171,230) (https://covid19.who.int/table).

That notwithstanding, it has been difficult to navigate the unending avalanche of apocalyptic declarations and unwarranted, definitive verdicts imposed on Africa since the beginning of the pandemic. All this, in unfortunate contrast to the realities unfolding on the continent. Consider the following headlines: “Africa’s pandemic puzzle: why so few cases and deaths?” (Nordling 2020); “The pandemic appears to have spared Africa so far. Scientists are struggling to explain why” (Science 2021); “Heavily unvaccinated Africa has so far avoided a COVID-19 disaster” (Associated Press 2021); “Coronavirus in Africa: Could poverty explain mystery of low death rate?” a BBC tweet (Sahara Reporters 2020) and “Scientists can’t explain puzzling lack of coronavirus outbreaks in Africa” (Smith 2020). It gets even ‘dystopically’ poignant with early attempts by formal organisations: A report by the United Nations Economic Commission for Africa (UNECA) in April 2020 had this prognosis about the whole continent: “Anywhere between 300,000 and 3.3 million African people could lose their lives as a direct result of COVID-19.” The above are some of the headlines from media coverage of COVID-19 in Africa in the past 2 years. To be fair, some are genuine science communications (Nordling 2020). However, many of them are from unsolicited, self-appointed experts who seek to save an Africa that is often deemed to have neither agency nor expertise — but seen only as a helpless victim.

These baseless speculations show colonial reflexes on full display during the crisis. The problem is that they miss the opportunity to be accurate, encouraging or nuanced. Consider further the following comments on live TV (CNN) by a prominent individual with heavy investments in vaccines: “It’s gonna be horrible in the developing world…Look at Ecuador, look at what’s going on in Ecuador. They are putting bodies out on the streets. You’re gonna see that in countries in Africa.” The prediction failed. Fast forward in 2022, as the pandemic wanes, the hope for an endemic stage as the Holy Grail for a return to normalcy was rekindled globally. Although Africa is the least vaccinated region, it still has the least mortality rates, least hospitalizations and most of the affected were asymptomatic or speedy recoverees. Asked about the world’s progress in ‘beating COVID-19’ during a panel at the 2022 Munich Security Conference, a rich tech businessman with heavy investments in vaccines (not a virologist or medical doctor) argued that: “Sadly, the virus itself—particularly the variant Omicron—is a type of vaccine. That is, it creates both B cell and T cell immunity. And it’s done a better job of getting out to the world population than we have with vaccines. Serosurvey in African countries is 80% positive (Hains 2022). The chance of severe disease is dramatically reduced because of that infection exposure. We didn’t do a good job on therapeutics.” Is this news to be celebrated or something to be sad about? Let the reader decipher.

While variations exist across the 54 countries (with South Africa being the most affected), the data show that the continent as a whole would have fared far worse with COVID-19, had it not been for its ‘rituals of self-determination’ (using the words of Gregg Carr) in public health governance and community focused crisis management. As a caveat, this paper is not based on country-level analyses but a regional level comparison. It juxtaposes the pandemic responses of African countries (using the example of Ghana) and Global North which is seen to have the highest level of health security in terms of innovations, infrastructure, expertise, and governance tools.

How have African countries been surviving since creation despite their exposure to numerous tropical diseases and recurrent epidemics? The answer is found in the neglected but rediscovered rituals of self-determination in public health through community-focused bottom up, preventive measures, frugal innovations (Harris et al. 2015; 2017a), and traditional and lately, modern therapeutics and prophylactics (Ahen and Salo-Ahen 2018). Epidemiological questions in the global South are extremely complex, and no single factor stands out as a hypothetical/empirical explanation (Wamai et al. 2021; WHO 2015). More prominently, it is not for journalists, IT gurus and people with little knowledge of the multifaceted African pharmacognosy regime to attempt to predict anything. It is worth noting that while their roles are scientifically inconclusive (but cannot be discounted), the environment (hot and humid temperatures) and youthful demographic structure, national health profiles (without predominance of obesity and other chronic diseases and a small percentage of over 65-year-olds) help account for the limited spread and severity of the virus (Diop et al. 2020; Wamai et al. 2021). In the beginning of the unfolding pandemic, the predictions for Africa were nothing short of apocalyptic. Without making any exceptions, the entire continent was portrayed as the face of poverty, having the highest disease burden, lacking health expertise and infrastructure and being the weakest in all performance matrices. For example, in the UN’s Human Development Index, African countries are in a never-changing bottom position. However, this time Africa defied the odds.

Those prophecies meant that Africans were supposed to claim victim status by default (CNN 2020). Consequently, as the default apocalyptic hypotheses were met with low death rates in Africa, that reality was challenged in many quarters with headlines such as “A Continent Where the Dead Are Not Counted (see for example, Maclean 2021). Implicit in that narrative is a longstanding belief system among various epistemic communities, global health governance, politics and agenda setting experts that the death, disease, and desperation must automatically be the narrative for Africa (Knoll and Ahen 2020). These are seen as immutable by-laws that govern all operations in global health diplomacy and scientific interpretations. Or are they? Such attitudes, however, negatively shape global health while maintaining the status quo and relevance of certain actors and agencies even though that undermines the Southern efforts. To be fair, Africa does not own the patent rights to be the face of misery and the eternal recipient of health resources and expertise from elsewhere while others get to be defined as the saviours even when they fail. Whether through frugal innovations (Harris et al. 2015) or sophisticated technologies and mitigation policies, on the whole Africa confronted COVID-19 with a fair wind of benefit from lessons from HIV/AIDS explosion that plagued it in the 1990s. Also, lessons from recurrent tropical disease epidemics and most recently the Ebola and other previous outbreaks (Mackey 2016; WHO 2015) as well as the use of foresighted strategies (Woods and Pannenborg 2021) have been useful.

## Managing Pandemics: Historical Foundations

For the past 300 years (since 1721) vaccines have progressively become recognised as the Holy Grail for battling pandemics albeit with massive resistance (Wilkerson 2020). Promising vaccines for COVID-19 have been administered on billions while several are still in the pipeline. This makes 2022 a year of rebound after a historically unprecedented pandemic. The current global health inequalities can be attributed to the pathology of the present global economy (Labonté 2017) just as much as that of global politics (Ottersen et al. 2014) and leadership (Ghebreyesus et al. 2018). The science and politics of global health in managing or mismanaging pandemics are in every way extensively predictable (Ahen 2021). Politics, corporate political power, and the agenda setting status of nations, international agencies and regional bodies in global health determine outcomes (Benatar 2017). However, as Ottersen et al. (2014) put it “in many cases the political origins of health inequity are either ignored or played down”. The two major forces that are fundamental determinants of health are poverty and inequality (Maclean et al. 2009). This inequality is more pronounced in the asymmetrical distribution of medico-techno-scientific innovations that are monopolized in the global North as a competitive advantage (Ahen 2021; Trindade Lima and Grabois Gadelha 2021). The consistent quest has been about public health governance reforms to provide access to quality medicines through protected supply chains, acceleration and updating of public health infrastructure, while guaranteeing improved socioeconomic determinants of health at the community level (Woods and Pannenborg 2021; Ghebreyesus et al. 2018). This is supposed to be the essence of sustainable global health as envisioned by the United Nations (UN) Sustainable Development Goals, SDGs; United Nations 2020). Regrettably, that is not what is seen, given the top-down approach that repeats the ‘manias’, consistent with the protection of the status quo while marginalizing Southern causes and public health management approaches (Ahen 2021). In what follows, I explain the emancipatory theoretic frame for this study followed by the political economy of vaccine development (vaccine nationalism) with a critical review of a Pan-Africanist alternative approach through rituals of self-determination in national and regional information coordination. This is achieved through the WHO, African Centres for Diseases Control and national health systems approaches to managing the pandemic. A panoramic vista of the situation in Africa is offered with an account of the case of Ghana. This is followed by the introduction to an ‘immune system’ framework, conclusions, and foresight points.

## An Emancipatory Theoretical Approach

This paper practices a combination of emancipatory theorising and decolonization of theories, policies and methods (Bhambra 2021) used in framing narratives about sustainable global health (United Nations 2020). Here, different forms of emancipatory approaches of theorising are employed to point to critical health ideals while questioning received wisdom and biased colonial views or systems of domination and how to not only subvert them, but also to prescribe ways in which they can be improved or replaced (Cornelissen et al. 2021). It changes the terms of the global health debate by embedding the analysis in the spirit of our times, and by owning the script of the narrative rather than being aloof consumers of one-sided epistemic inputs. The expected outcome is independence or interdependence between nations rather than top-down paternalistic approach or dependency on global health ‘governors’. Emancipatory theorising as applied here challenges the unmutated view of Africa as incapable and always in need of salvation. Despite the new data/empirical evidence, many positive news from Africa come under hostile scrutiny and official disdain. According to 2018 Ibrahim Index of African Governance (http://mo.ibrahim.foundation) good governance across the continent has increased. And in 2020 African nations were able to mitigate the COVID-19 using approaches that even nations with the *highest global health security* could not match such performance in the beginning (Khan 2020).

Emancipatory theorising is about “revealing the structures of domination and human constraints that are inscribed into our current beliefs (which may variably be expressed as suppressed forms of consciousness, ideas, discourses, or bodily behaviours), and by trying to make a real, practical difference through identifying the potentialities and possibilities for emancipation and reform” (Cornelissen et al. 2021:12). Whereas interpretive theorising focuses on “theoretical abstraction and synthesis”, emancipatory theorizing is “bent on using theory” (ibid) as an opportunity to shine a light on normative and practical issues of inconsistencies, biases, accepted but unproductive protocols and ideological frameworks surrounding narratives. The objective here is to provide avenues for change to the status quo which by default discredits and downplays good news from Africa. This approach takes the form of ‘intellectual activism’ in the tradition of Angelia Y. Davis (Davis 2016) and Antonio Gramsci (Gramsci 1971). It is a progressive academic praxis “or a particular type of critical performativity to help scholars make a difference in the world” (Contu 2020:1). It calls on scholars to engage with the challenges posed by emerging crisis that affect society on a structural level. These include socioeconomic, political, and environmental justice issues (Contu, 2020). The works of Alvesson and Sandberg (2011) and Banerjee and Arjaliès (2021) are other examples of the meta-theoretical critique employed here to problematize, question, expose, and challenge powerful systems of domination. In this process the researcher does not pretend any more (as originally socialized to be detached from the object of study). Rather, he or she becomes an active agent and a crucial part of the discourse and not a bystander (Deetz 1996). The objective is to imbue new forms of meaning into a discourse and to disrupt the status quo through the creation of new realities with new theories in seeking epistemic and substantive justice (Bhambra 2021). It is about boosting what is conceptually, instrumentally and legitimately relevant/impactful in a defined context (Nicolai and Seidl 2010) instead of pretending that there are no self-interests or foreign policy agendas that profit from crises—whether they are wars or pandemics.

Hence, in the neo-liberal era of weaponized fake news and conspiracy theories, we take every word with a pinch of salt since “the apparatus of propaganda in favour of corporate interests” [knows no boundaries] (Higgs 2017:15) emphasis added. I endorse the epistomological and ontological position that “...changing reality requires producing other types of knowledge. Limiting ourselves to condemning and rejecting extant views paradoxically risks the reproduction of the hegemonic reality we intend to question” (Janssens and Zanoni 2021:3). Moreover, existing health problems have intersected with the COVID-19 pandemic, which has further compounded long-standing health and economic disparities between the global North and the global South and even the peripheries within the centres. Thus, minorities/low-income households in the US and UK, or Brazil, for example, are the most affected by COVID-19 (Centers for Diseases Control and Prevention, CDC, USA).

## The Political Economy of Health Inequality: Vaccine Nationalism, Medical Apartheid, or Vaccine Gold Rush?

In the 1990s, Big Pharma instituted legal proceedings against the government of South Africa during the HIV/AIDs epidemic. The government wanted to make cheaper generic versions of anti-retroviral drugs, but the industry opposed that. This idea of putting profits before humans prompted protests around the world. The industry and Northern governments’ actions were clearly the antithesis of what sustainable global health needs to be. There are several examples of denied or delayed responses even during serious epidemics.

In the current pandemic, rich nations ordered the vaccines well in advance before there even was licensing, in order to guarantee their access to the first batches (Torjesen, 2020). Per a Financial Times report, the US government had a deal to spend $1.95bn (£1.49bn; €1.65bn) on 100 million doses of BioNTech/Pfizer’s candidate vaccine. Vaccine nationalism then is seen in the US, formally abandoning the WHO, and embarking on an ‘America first’ policy that ‘selfishly’ prioritizes sufficient supply of emergency use (Food and Drug Administration, FDA) approved vaccines and therapeutics for use in the United States. Although some wealthy nations condemned such inward looking interventions, they nevertheless adopted a part of it by ordering their own vaccines in advance. Such behaviours have become known as ‘vaccine nationalism’ (Eaton 2021).

The old problems persisted despite the COVAX initiative to provide low-income nations with equitable access to vaccines (WHO 2020). They consist of what Ahen (2021) refers to as ultimate preference for non-optimal solutions in global health governance. These include the concentration of medico-techno-scientific resources in industrialized nations and the creation of a system of dependency where the global South must survive mainly at the mercies of donations. According to this theory,’for any given set of global health solutions for creating value or prevention of public health governance failures, a range of market and institutional possibilities always exist. Nevertheless, the data show that deliberate quick fixes or delayed interventions as seen during COVID-19 are mostly preferred to sustainable options. This allows the major actors to maintain the status quo (relevance/survival-seeking) and the attendant incentive structures—leading to weak governance structures that undermine the sustainability and institutionalization of global health as a major concern.’ Additionally, the theory explains why medico-techno-scientific products monopolized in the global North remain geopolitical commodities through which powerful actors leverage competitive advantage over the global South (Ahen 2021). This ensures the survival of actors (such as the pharmaceutical industry) and the maintenance of the power and profits of their communities of practice (Ahen 2021). Medical racism, biopolitics and biopower (Wilkerson 2020) clearly stood out as the core foundations of global health inequality during the pandemic (Espina and Narruhn 2021).

A typical example of ultimate preference for non-optimal solutions is vaccine nationalism. It is neither new nor different from medical apartheid (Washington 2006) or economic protectionism. These practices have existed since colonial times to keep control of resources for one group while depriving others. The disenfranchised are the last to receive care. Nevertheless, they are better used for medical experiments and when the real products are out, they are the last to receive them (Ghosh 2021; Shah 2010; Goldacre 2012; Fortunato 2021; Gherke 2012), given their status as distal stakeholders (Ahen 2017).

Moreover, Ghosh (2021) explains disaster capitalism with the example of India’s proposed opening of vaccination to the 18–45 age group from the beginning of May. This will however have two key restrictive attributes: (i) limited to private hospitals and clinics; (ii) based on cash and carry—only on payment—₹1,200 to ₹2,400 (€13.25-€26.5) for each dose. Thus, the lower income households are welcome if they can pay for such vaccines and if they cannot, its rather unfortunate. Such practice is what Ghosh refers to as “the privileging of corporate profits over human lives which marks our still-neoliberal world” (Ghosh 2021). This may seem cold and inhumane. However, the world is a business in which humanistic management takes a second place. Hence the need for decolonization.

The lack of access to medicine is a longstanding problem in developing nations (Ahen and Salo-Ahen 2018). It is part of crisis nationalism; a situation in which countries especially entitled, if not obliged, are to prioritize the interests or well-being of their own citizens during a global crisis, such as a global pandemic (Beaton et al. 2021). Beaton et al. (2021) conclude that while this may have an initial moral and practical justification, vaccine hoarding during global crises exceeds the justifiable limits of crisis nationalism. The practice of vaccine nationalism that deprives others of scarce resources (Eaton 2021) is a myopic worldview that prioritizes ‘our own’ people/nationals first, then hopefully the others. However, this is rational or model-based foolishness because “no one is safe until everyone is safe”. On the surface, it tells how rich and powerful nations play a hegemonic role in managing the global pandemic (Ahen 2021).

The discussions that follow offer an elaborative account with illustrative examples of ultimate preference for non-optimal solutions. The objective here is to avoid the semblance of leaps of inference on the part of readers who may not be steeped in critical studies.

## Patent Rights or Human Lives?: The Antithesis to a Humanistic Management Perspective

Fortunato (2021), citing Fiercepharma.com explains how BioNTech and Curvac received 745 million from the German Ministry of Education and Research. Prior to that another 20 million in concessionary lending had already been given by the European Investment Bank (Knight 2020). Pfizer for example received 2billion from the US government as down payment for 100 million doses vaccine. This was all meant to be a form of risk sharing that is inherent in the complex and risky process of vaccine development. Vaccine research is dependent on public investments, basic science from public universities and public/institutional services for approval, clinical trials and final approvals or emergency approvals for pandemics (Fortunato 2021).

What’s more, AstraZeneca punctually arrived on the scene only after a “vaccine developed by Oxford University—almost wholly publicly funded—entered the phase of clinical testing and production, just as with Pfizer” (Fortunato 2021). Notwithstanding the significant public contribution, as Fortunato (2021) argues, Big Pharma can claim “exclusive intellectual property rights and accruing massive profits”. And Pfizer, along with the rest of the industry, has been lobbying to stop a temporary waiver of intellectual property rights (IPR) (via patents, copyrights, and trademarks), endorsed by the current US administration to allow generic COVID-19 vaccines to be distributed at low cost in the global South under the auspices of COVAX (Ghosh 2021). Instead, as usual, the industry is claiming monopoly rights in all those developing economies that ratified the agreement on Trade-Related Aspects of Intellectual Property Rights (TRIPS), which came into force back in 1995— despite the awareness that COVID-19 is a global scourge. As usual, changes to this is almost an insurmountable task due to intense and incessant lobbying and regulatory capture by Big Pharma to attain unjustifiable profits (Goldacre 2012; Shah, 2010). Additionally, Fortunato (2021) refers to two well-known disturbing practices inherent in the industry: (i) ‘patent thickets’ (overlapping patents covering a wide area of economic activity and potential downstream inventions) and ‘patent fencing’ (excessive patenting with a view to cordoning off areas of future research)”. The above behaviours by a cartel of major actors ignited the rituals of self-determination as in the case of Ghana.

## The Case of Ghana: The Favourable Extrinsic and Intrinsic Public Health Factors

Africa had a few advantages that other regions didn’t have. (i) Age – According to Worldometre, as of June 2022, Africa’s population stood at 1,403,066,538 with a median age of 19.7 years representing the youngest in the world^1^. Younger people tend to have a strong immune system compared to elderly people. (ii) Spatial dispersion of settlement and the interactions between much younger and elderly compared to Western nations. Here, the elderly are placed in hospices where widespread infection is commonplace. Mukherjee (2021) argues that that there are both intrinsic vulnerabilities (such as age or pre-existing conditions) and extrinsic vulnerabilities (the structures of households, the rate of interpersonal contact in residence). Putting all these factors together, models of COVID-19 mortality predictions failed by wide margins on developing nations. Most of those who contracted COVID-19 were asymptomatic or mildly asymptomatic and they quickly ‘recovered’; thus, tested negative and life went on normally. “Nigeria was predicted to have between two hundred thousand and four hundred and eighteen thousand *COVID*-19 deaths; the number reported in 2020 was under thirteen hundred. Ghana, with some thirty million residents, was predicted to see as many as seventy-five thousand deaths; the number reported in 2020 was a little more than three hundred” (Mukherjee 2021).

The Africa Centres for Diseases Control quickly established Africa Taskforce for Coronavirus (AFCOR) on February 5, 2020. In Ghana, where I was located at the time in 2020, it was soon discovered that they saved millions from importation of rice and other food stuffs (www.ghanagov.gh) that were locally available but under preferred to imported foodstuffs. In almost all the speeches by President Nana Akufo-Addo, he mentioned specific African foods that boost the immune system. Medical doctors, nutritionists and others filled the airwaves with their best advice for the population (The Presidency of Ghana 2020, 2021). Additionally, a law was enacted to curb false information, misinformation, disinformation, fake news, and conspiracy theories about the outbreak. This ensured that all claims were not politically influenced or made by charlatans who may sell non-approved COVID-19 medicines for profits. Science-based information was prioritized along with the crucial role of the (mostly people-centered) independent media. The prioritization of incentive structures for frontline workers served as added impetus for excellent service and a better patient care. This is because research has found that non-payment and underpayment of clinicians and health workers especially aggravates health inequalities (Coffey et al. 2020). The attitudinal change led to new hyper hygienic health practices as the new normal. On the economic front, consumption of locally produced foods and provision of free food, drinkable water and subsidized electricity for urban poor was executed (www.ghanagov.gh).

The institutional heterogeneity of African countries does not warrant one-size-fits-all solutions. However, certain rules of mutual engagement were developed. Principally, the African CDC, the Noguchi Centre for Scientific Research and the Ministry of Health consistently provided health updates across media platforms. The local seamstresses and apparel firms diversified into mask production. Alcohol distilling companies became producers of hand sanitizers with the FDA closely scrutinizing and giving speedy approval. “No mask, no entry” policies were enforced in all shops selling essentials. Tracking, tracing, and information guidelines were extensively made available. Schools and Universities closed. the citizens mainly complied because police and military were deployed to ensure strict compliance with COVID regulations in Accra where the few recorded cases were predominant. Only essential workers could operate. None of these non-pharmaceutical measures required external intervention and yet across most of Africa the countries that were deemed less susceptible (Egypt and South Africa because they had better healthcare) were still among the most affected. In effect, the contagion was significantly slowed, lockdowns were eased and even the few hundreds that were affected were very mild (Wamai et al. 2021). The lockdowns were a contagion control, approach that helped in curbing superspreading events such as parties and nightlife (Althouse et al. 2020). The prophesied sight of dying Africans, scattered in the streets and in mass graves did not happen in 2020 nor in 2021. Rather, Africa must grapple with its old problems. Malaria and other prevalent tropical diseases, not COVID-19.

## The Historicity of Rituals of Self-Determination in Public Health Interventions

Africa’s dependence on others for their public health resources and expertise is a new phenomenon. Back in 1721 ‘variolation’ (referred to at the time as ‘inoculation’) became a thing from Africa to the US. Here, “pus is taken from a smallpox blister and introduced into a scratch in the skin of an uninfected person to confer protection”. This knowledge was brought to New England by a West African (Liberian) slave by name Onesimus in 1721. After Onesimus explained the tricks and procedures to a Boston doctor/Minister Cotton Mather, the doctor decided to experiment it during a smallpox outbreak—saving dozens of lives. A couple of decades later the newfound cure spread beyond Massachusetts to become the standard practice in the US. Then as now, it was not without heavy resistance. It is on the basis of this discovery that modern scientific procedures for producing vaccinations were built (Wilkerson 2020). This is just one example that shows that Africa has existed with its idea of cure without dependence long before anyone showed up with ideas of disease management for profit.

Global health misgovernance promotes inequalities with massive tolls on human lives. This is the origin of the global South’s severe discontent (Wilkerson 2020; Ahen 2021). Before the international regulation on health in 1851 was converted into WHO in 1948, black Africans were not deemed fully human, or if they were, they were considered very much less than other groups. They were useful for slavery and objectified in scientific studies, operated on without anaesthetics or painkillers, or left to suffer as guinea pigs (Wilkerson 2020; Washington 2006). See the Tuskegee experiments on over 400 black men in Washington 2006). Consequently, numerous cases of clinical trials without informed consent that resulted in deaths have been conducted on the poor across Africa (Shah 2010; Goldacre 2012). When COVID-19 started, the world counted Africa out before spuriously deciding it was something else that accounted for her success in managing the pandemic. Rituals of self-determination as applied here in public/global heath governance are the full gamut of spontaneous, contextually relevant self-initiated practices, policies, frugal innovations, and traditional therapeutics employed in enhancing public/global health issues. Rituals of self-determination consist of medico-techno-scientific and non-pharmaceutical mitigation strategies that are institutionalized by emergency (like a war time mass behaviour) and conditioned by circumstances. The foundational axioms of (rituals of self-determination or public policy interventions are articulated as follows in Table 1:

**Table 1:** Components, characteristics, and scope of rituals of self determination

Components of the rituals	Characterization
Strong leadership and political will for enacting change	The first step in the practice of rituals of self-determination is a strong leadership (with political will) and a clear vision with a united front
Employing tried and tested methods from grassroots	The second is the acknowledgement of frugal innovations, common sense policies and by making public health questions apolitical
Self-sufficiency / non-dependency	These rituals stem from the realization of a sense of urgency and the sacred duty to ensure that the survival of communities and nations during a public health crisis depends on themselves first, and international support second (if any)
Proactive & Pre-emptive interventions	These public health practices are employed when nations acknowledge the deficiencies in their own existing healthcare systems and efficiently use available albeit limited resources to mitigate the crisis rather than trying to solve it when it has become insurmountable
Decolonialized public health governance	Autonomy and agency: Thinking and doing for self. The rituals are solidified into institutions due to historical indignities suffered by the global South. These include medical racism, scientific racism, testing of drugs on innocent people without informed consent (Wilkerson 2020; Washington 2016) and the recent medical racism as a public health crisis laid bare by the pandemic (WHO 2021; Bailey and Moon (2020);
Institutionalized mistrust	The intensity of the rituals manifest when nations can no longer count on the assistance of the rich nations (at least in the immediate to medium term) but are also unsure when and how such support will arrive and under what terms. Moreover, the rituals are performed without waiting for traditional benefactors or experts
Urgent Stock taking	Rituals involve an immediate valuing of contextually neglected frugal innovations, resources and capitalizing on them to create useful public health outcomes
Recourse to the basics	Investments in the most important social determinants of health: sanitation, nutrition, and safe-drinking water along with reduction in pollution levels before considering vaccines as a last resort. Like most African countries, Ghana embarked on all the above interventions

In contrast to dependency on global health governors, donors, international cooperation and aid agencies, the rituals of self-determination’s connection to decolonization lies in how governments take full responsibility in times of emergency. They rely on past experiences with similar outbreaks. They recognise the need to decolonize cure and public health management while making the most of common sense and cost-effective strategies to save lives in ways that are institutionally and contextually suitable. Here is a synthesis of explicit and implicit strategies that were employed across the continent:

1. Africa showed foresight with the benefit of historical hindsight. This was based on the historically documented fact that the rest of the world would normally prioritize their own population before offering any support to Africa. This happened during COVID-19 as it did during the Ebola epidemic.
2. Effecting quick lockdowns and travel restrictions meant easy containment in countries and between countries (Wells et al. 2020).
3. There was a cohesive intra-African coordination and information sharing with the Africa CDC and national ministries of health https://africacdc.org/download/annual-progress-report-2020/. For example, immediate support for low-income urban residents with food supply and free water and electricity in Ghana changed the trajectory of potentially ‘wicked’ outcomes.
4. More progress was made with effective and urgent mechanisms for information diffusion to quell superstitions with science-based narratives and coordinated hyper-hygienic public behaviour.
5. Intracontinental and regional coordination supported isolated and incoherent national strategies for adaptation to new protocols in border security and pandemic response capacity building.

The above governance mechanisms work by persuading the masses that these interventions are shaped by rational decisions for guaranteeing health as a national biosecurity concern. The rituals also include tried and tested approaches such as fixing social determinants of health and the employment of availability heuristics where policy guidelines are upgraded as authorities receive more and better understanding of the situation as it unfolds. The rituals are both systemic and community/grassroots (Woods and Pannenborg 2021) and individual responsibility. Additionally, the strong and active state-community leadership can also be explained by the ‘elite panic’ as further explained.

## Theoretical Explanations: How ‘Elite Panic’ Forces the Urgency for a Return to Rituals

Although the global South lack a strong financial base to completely overhaul their weak health systems, proper governance during the pandemic led to a prompt intervention rather than the usual empty rhetoric. In normal times, however, this does not happen until governments’ hands are forced. Typically, the ‘elite panic’ occurs when those at the helm of affairs are spurred into action because of their own distress (Solnit 2010). Here, the fear that the population will panic, leading to uncontrollable disturbances/an insurrection that will eventually threaten the established order is the main impetus for action rather than solidarity or altruism. On the contrary, citizens embark on extraordinary community-based solidarity (WHO 2015; Mackey 2016; Moon et al. 2015) rather than expected chaos and selfishness (Solnit 2010). The handling of the pandemic in many African countries through spontaneous government-community relations was a watershed moment that showed new rituals of self-determination in renegotiating their place in global health governance. The rituals were also pushed by four factors that are normally associated with the elite panic during the pandemic.

First, at the onset of the outbreak, there were myriad political and pseudoscientific predictions about Africa. However, when the forecasts failed to materialize, instead of looking at the key success factors and their antecedents, some scholars and commentators attributed it to the non-availability of trustworthy data (Wamai et al., 2021). They ignored the efforts of African governments, ministries of health, the African CDC and a return to bottom-up community-based health and information sharing (Woods and Pannenborg 2021).

Second, just like HIV/AIDS (which was first discovered in New York in 1981 and never recorded in Africa until 1983; Versi 1990), the SARS-CoV-2 virus did not come from Africa (WHO 2021) and African governments and peoples wanted to keep it at that because it matters for their reputation. Rather, the strange pneumonia was first reported from Wuhan, Hubei province of China (Zhou et al. 2020; Wu, et al. 2020). The non-African origin of COVID-19 is also corroborated by for example the British Professor Angus Dalgleish and Norwegian scientist Dr Birger Sorensen who “wrote that the virus had no credible ‘natural ancestor’ and argued that it was ‘beyond reasonable doubt’ that the disease was produced through ‘laboratory manipulation’” (Owen 2021). Even Facebook had to reverse its ban on news about the virus coming from elsewhere. “In light of ongoing investigations into the origin of COVID-19 and in consultation with public health experts, we will no longer remove the claim that COVID-19 is man-made or manufactured from our apps, Facebook announced…” (Powell 2021).

Even so, the non-African origin of the virus matters because it removes one more stigma from the existing pile of existing stigmas such as Ebola (Mackey 2016) and the negative branding associated with Africa. Serious rituals of self-determination helped in a reputational bounce. In terms of socioeconomic determinants of health, reports of worsening public health in Africa affects business, economic development and the attraction of foreign direct investments that creates jobs and prosperity and better living standards (Ahen 2021). In addition, the negative image and consequent treatment of Africans by way of racism, discrimination, lack of opportunities and education, health and employment and income inequalities have all become another global pandemic that hits Africans at the cellular level. These cause early death, cardiovascular diseases, and mental health problems according to the CDC. These underlying problems have made African Americans and other Africans more susceptible to the pandemic in the UK and the US (Hughes et al. 2021). Apart from the global pandemic, Africa and her diaspora have had to confront equally urgent questions with racism (The Guardian 2020). It is for this reason that governments in Africa did not relent in their efforts to take broad-based non-pharmaceutical measures in handling the outbreak. Least not because they had a serious public health infrastructural and human resource deficit compared to other regions, but also because they are always put last in global health governance as in vaccine nationalism (Ghosh 2021; Beaton et al. 2021; Eaton 2021).

Third, when the first African cases of COVID-19 were detected in Egypt on 14^th^ February 2020 via travellers from Europe (Mehtar et al., 2020), the rest of Africa quickly moved to contain it better than most regions of the world; specifically, Europe, the US and Brazil where there was a lot of denial. The exceptional cases were that of Tanzania, which ignored testing protocols, and that of Eswatini, which even started lockdowns before a single case was registered. Mauritius also proposed a herbal concoction. Before COVID-19, three West African nations (Sierra Leone, Liberia, and Guinea) suffered from the Ebola outbreak that killed several thousand people, but their neighbours vigorously and competently fought it off with science-based interventions (WHO 2015). In hindsight, it is worth acknowledging how capable Africa is, as Nigeria, Senegal and Mali fended off Ebola in 2014 but received less global recognition. This emphasizes the fact that many nations in the West African sub-region already had ‘under their sleeves’ pandemic response infrastructure and strategies in place given previous experience from the Ebola outbreak. Any threat of an outbreak was never taken lightly across the continent because the most important lesson from Ebola was that it would get worse before global actors step in. Therefore, countries must act proactively in self-defence.

Fourth, Africa’s frontline workers were the easily forgotten heroes. Also, philanthropists such as Nigeria’s billionaire Aliko Dangote and others contributed massive amounts but received poor media attention (www.business-humanrights.org). Embedded in the rituals of self-determination is a new sense of responsibility “by us for us”. However, none of such strong displays of political will received any high-profile media attention.

African governments approached COVID-19 in a traditional fashion through prevention and respect for the virus and the political will to enforce those emergency protocols. Whether it is viewed as premature exuberance or a minor breakthrough, it is worth recognising the successes of African governments in their COVID-19 responses for two reasons. One because this was not the expectation of global experts and two because it made a big difference in saving lives. This latter point is a most laudable humanistic approach because it promotes human wellbeing and dignity (Pirson, 2019) which is the raison d’être of public services to people (Ruffini et al. 2022). The end goal is to reclaim people’s humanity (Pirson, 2018) even in perilous times.

## How did Nations with Highest Global Health Security Respond?

On the contrary, across the West, the knee-jerk responses to the pandemic were palpable, coupled with loathing of lessons from Africa’s experience as was the case of Ebola (Mackey 2016; WHO 2015). Most Western countries (except for Scandinavian countries, although Sweden took a different stance) lackadaisically approached the crisis. Some even suggested that it is “a thing for the third world”, “just a flu” and were even late in providing personal protection equipment for clinicians and oxygen for patients. — This led to high mortalities among the frontline workers. African countries did not underestimate the crisis. This is because they were acutely aware of the lack of health infrastructure and the inadequacy of the health system to accommodate the emerging demands coupled with the existing diseases burden (Wamai et al. 2021).

The world failed to learn from Africa’s experience, and it bore the brunt. For example, when the WHO strongly recommended the nations to ‘test, test, test”, the deputy chief medical officer of England Dr Jenny Harries vehemently argued that the WHO guidelines did not apply to Britain’s “extremely well-developed public health system” …that they were meant more for “low income” countries (Kara and Khan 2020). Even in the face of a non-discriminatory virus, these colonial views and attitudes implicitly forced themselves into national policies where lessons cannot be taken from the periphery because knowledge flows strictly and only from the global North to the global South and not vice-versa (Dörrenbächer et al. 2021). Such hubris and cruel overconfidence came with huge cost, and by April 2020 there was an about turn towards, “testing”, “testing” and more “testing” on the knees before a potent virus which was no respecter of the fictional first world or third world geopolitical binaries.

Dr. Mishal Khan writing in the Telegragh puts it clearly that “The strengths of high-income countries are overstated when it comes to pandemic preparedness” (The Telegragh, 2020). Most notably, Ms Zampa, the Italian undersecretary for health once famously suggested during the pandemic about Italy’s slow reaction that “Italy looked at the example of China not as a practical warning, but as a ‘science fiction movie that had nothing to do with us.” And when the virus exploded, “Europe”, she said, “looked at us the same way we looked at China.” (New York Times 2020). However, the distrust, disrespect, and rejection of knowledge from the global South is neither shocking nor new. Harris et al. (2015; 2017a, b) from the Imperial College have done several research studies on the biases in review, acceptance or even recognition of publications or health innovations from developing nations for policy making in the West. As Harris put it, “they hear Africa, and they think there can’t be any good services (Harris et al. 2015; Labonté 2020). In their 2017 study using implicit association tests, it was concluded that most scientific studies are associated with country of origin (Martin et al. 2011). Thus, good research by default comes from rich countries and bad research from poor countries, thereby blocking the diffusion of health innovations and excellent ideas that could have been useful had they been adopted. However, there is something else amiss that is much more sinister than the biases. There is a complicated tension between the global North and the global South regarding the superiority complex that leads people away from giving recognition to those it is due because the low-income nations also happen to be those that were colonised. Thus, exposure to the country of origin of an idea or product or person may in many cases trigger biases and stereotypes (Martin et al. 2011). This explains the low representation of low-income setting scholars in medical journals (Tutarel 2004).

In sum, despite the third wave in many African nations, the continent is the least affected region. According to the WHO, despite the many forecasts that predicted doom on the horizon (El-Sadr and Justman 2020). That is not a call for premature exuberance or encouragement towards complacency. Rather, it is to suggest that proactive leadership, low-cost preventive measures (Fokoua-Maxime et al. 2020) in public health and the advantage of historical hindsight from previous outbreak such as Ebola, when taken seriously, can produce actual results in terms of saved lives and decreased infections (Ahen 2021). Africa’s success is less likely to be highlighted but rather put in doubt (Fokoua-Maxime et al. 2020; Dzinamarira et al. 2020). This is also not to obscure the fact that during the period of the pandemic, malaria has caused more mortalities and morbidities than COVID-19 in Africa (Kelland 2020). How could experts possibly trust this data but not trust that COVID-19 impacted Africa less due to proper grassroots management and other favourable demographic, environmental and settlement factors? Moreover, the success in containing COVID-19 is not an attempt to sugar-coat the weak socio-economic determinants of health, chronic lack of access to medicines and health infrastructure, and the intermittent financing of health (Ahen and Salo-Ahen 2018). However, precisely these issues have forced African leaders to rethink their approaches to sustainable global health via non-pharmaceutical and mitigation approaches.

## Introducing the ‘Immune System’ Framework for Public Health Governance

**Fig. 1:**
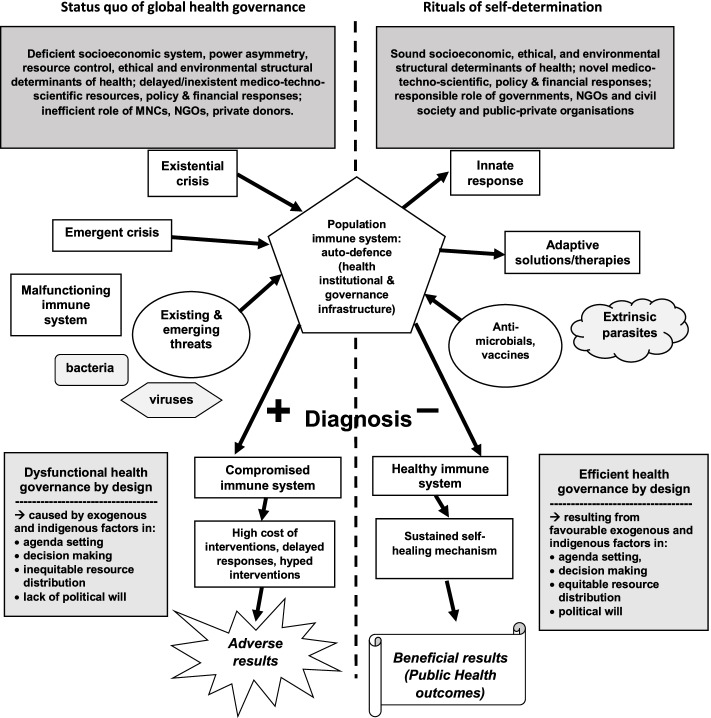
Population health infrastructure as an immune system reacting to external stimuli

In this penultimate section, I provide a framework for understanding global health interventions that are based on a simple cause and effect. The causes comprise moral, political, scientific, and community-based medico-techno-scientific resources acting as the ‘immune system’ and the effect—non/optimal outcomes. The weaker the immune system is, the worse the outcome will be and the stronger the emphasis on these are, the better the outcome will be in fixing dangerous public health problems. The cause and effects here are clear, positive results or negative results. Objectively and meaningfully, we can categorically sum up the various variables by interrogating and framing the global health narrative and outcomes it produces. This non-dependence of the centrality (absoluteness) of global health governors and their top-down policies (as against rituals of self-determination) produces immediate and pronounced outcomes as described in Fig. 1 below:

## Immune System: Historical Hindsight and Ritualistic Lessons for Foresight

The above model provides a clear line between positive (compromised) and negative (healthy) diagnosis of a public health system. The dotted line demonstrates a clear demarcation between the right and left sides of the model. If the 2014 Ebola outbreak taught African nations any lessons that could be taken seriously by policy makers and entrepreneurs/innovators, it is that the next disease outbreak would be overwhelmingly burdensome, surprisingly uncontainable and the human and economic cost would be incalculable. Africa’s low COVID-19 death rate still puzzles and causes confusion in some quarters because this is simply the merit of community level cooperation with leaders. Experts’ expectations of Africa compared to the rest of world was nothing short of dismal. In what follows I outline some of the major lessons learnt from the management of the pandemic in Africa.

First, the many deaths in low-income communities in the US and UK exposed the health inequalities and poverty, combined with pre-existing conditions such as obesity or chronic respiratory diseases. These problems made people more susceptible to COVID-19. The second lesson is political. The nature of the pandemic gave no space for the politicisation of public health in Africa. There was citizen-government-community level mutual respect towards the crisis. They have witnessed it so close to home many times and it was not taken lightly.

Third, since international commerce halted, nations looked within to sell and consume what they produced. That added to household income besides eating immune boosting organic foods that are health promoting. These plant-based diets have a bearing on strong immunity compared to fancy processed foods and less nutritious imported white rice, for example. This finally became Africa’s competitive advantage. This is a lesson to be taken because it reinforces the validity of this model. Fourth, many neighbouring countries employed various non-pharmaceutical interventions such as border control and quarantining, testing, and others to stop the contagion from spreading to neighbouring nations. This was a teachable moment that primarily accounted for the urgency with which African nations addressed COVID-19. Fifth, the three nations previously affected by Ebola became almost paralysed as international support for whatever reason delayed until the TVs were filled with gory images of the morbidities and mortalities. African governments did not want a repetition of such a scenario.

Sixth, besides Western allopathic medicines, it is widespread knowledge that traditional medicines or herbal medicines have the potential to boost the immune system and that they are widely used across Africa, something that requires more investments to spread its benefits (Addae Mensah et al., 2011). A seventh noteworthy point is that across the global South, there were no restrictions on or the demonization of the alternative use of some prophylactics such as ivermectin. Many studies show that regular use of ivermectin as a prophylactic was associated with significantly reduced COVID-19 infection, hospitalization, and mortality rates (Kerr et al. 2022).

In synthesis, it is precisely these lessons of being possibly left alone that moved African nations to rapid action. Through non-pharmaceutical interventions and prevention and community-based approaches (WHO 2015; Mackey 2016; Harvard-LSHTM). COVID-19 ushered Africa into a renewed hyper-hygienic regime where handwashing in schools, offices and shopping centres has become a norm at least in urban areas where the situation was concentrated. Villagers or those living in sparsely populated areas had that advantage that their immune systems have adapted to fighting certain diseases and infections due to chronic exposure to multiple germs—this has been called the ‘hygiene hypothesis’ (Wamai et al. 2021).

## Conclusions and Future Outlook

In the beginning of the COVID-19 outbreak, a predictable consensus was shaped around what the impact would be on Africa. Some self-appointed experts and non-experts argued that Africa will be the hardest hit region. Nevertheless, the experts and prophets of doom were wrong about Africa’s political will and their governments’ ability to respond competently to COVID-19 using frugal innovations and non-pharmaceutical approaches. Some attributed Africa’s success to the stars, environmental factors, or invisible agents but not the competence, intelligence, and humility of the African health intelligentsia in confronting what looked like an insurmountable crisis for other nations that were deemed ‘highly prepared.’

This paper argues that it took more than material medico-techno-scientific resources to handle the pandemic. The crucial role of human-centered leadership, historical hindsight and the trust between governments and communities at the grassroots level became the deal breakers in reducing the impact of the outbreak as in rituals of self-determination in public health governance. I further argue that while traditional cooperation is necessary for resources and expertise from the global North on big questions of sustainable health, the political will of Southern governments remains fundamental to any extraordinary success due to its grassroots orientation. Therefore, I maintain that continental Africa can no longer be necessarily defined by troubles, calamities, incompetence, or eternal victimhood but by its ability to competently handle complex and systemic issues through proper governance if there is a political will and minimal external interference. A novelty in this paper is that it highlights the fact that Africans are the only ones who can redeem themselves and there is the need to build on the three pillars of leadership, prevention, and decolonized science-based approaches to self-determination in public health governance that is also sufficiently foresighted to invest heavily in infrastructure. There is a need for a paradigm shift towards sustainable pharmaceutical innovations to be locally owned and defined as a form of resistance to the status quo (Callaghan 2019). There is also the need for a return to the 1978 “Health for all by the year 2000” primary health care initiative—popularly known as the Alma-Ata declaration. Health is personal, community-based and cannot be all dictated top-down, while ignoring the grassroots (Woods and Pannenborg 2021). The lessons show that Africa’s health issues can no longer be outsourced to experts who lack a profound understanding of the contextual realities. As Ghebreyesus et al. (2018) argues, this great Alma-Ata vision was undermined by “a lack of political leadership and economic crises. However, those conditions are no longer the case today in most African countries with thriving democracies. There are still many promising lines of enquiry to be pursued if global health in the South will have sustainable futures. Attention must therefore be paid to the rituals of self-determination and lessons must be learnt from Africa’s approaches to encourage decolonized governance in global public health.

## Footnotes

1 (https://www.worldometers.info/world-population/africa-population/)
